# Conceptualizing and Measuring Mindfulness in School Settings: A Mixed Methods Study and Development of Mindfulness in School Scale †

**DOI:** 10.3390/jintelligence14090210

**Published:** 2026-09-01

**Authors:** Ziyaeddin Halid İpek, Ferudun Sezgin

**Affiliations:** 1 Department of Educational Sciences, Kafkas University, Kars 36100, Turkey; 2 Department of Educational Sciences, Gazi University, Ankara 06500, Turkey; ferudun@gazi.edu.tr

**Keywords:** mindfulness, teachers, emotional intelligence, Mindfulness in School Scale (MSS), psychometric validation, exploratory sequential mixed methods

## Abstract

This study aimed to explore how mindfulness is perceived and experienced by teachers and to develop and validate the Mindfulness in School Scale (MSS) for use in school settings. An exploratory sequential mixed methods design was employed. In the qualitative phase, phenomenological interviews were conducted with 23 teachers selected through purposive maximum variation sampling. Content analysis identified six themes: Self-Observation, Environmental Observation, Self-Awareness, Environmental Awareness, Acceptance, and Reactive Control. These findings informed the development of the MSS. In the quantitative phase, the scale was administered to 663 teachers. Exploratory and Confirmatory Factor Analyses supported a five-dimensional structure comprising Self-Observation, Environmental Observation, Self-Awareness, Environmental Awareness, and Acceptance, explaining 51.46% of the total variance. Standardized CFA factor loadings ranged from 0.50 to 0.90, and the measurement model demonstrated satisfactory fit (χ^2^/df = 1.20, RMSEA = 0.028, CFI = 0.984, TLI = 0.981). The five dimensions also demonstrated satisfactory internal consistency, with Cronbach’s alpha coefficients ranging from 0.80 to 0.93. Correlations among the dimensions were generally low to moderate, indicating that the MSS captures related but distinct aspects of mindfulness in school settings rather than a single underlying construct. The findings further suggest that mindfulness in schools is shaped by both intrapersonal and interpersonal processes, with environmental awareness emerging as a contextually salient dimension. Overall, the MSS provides initial evidence for the validity and reliability of its five dimensions as a multidimensional instrument for assessing mindfulness among teachers in school settings while offering a context-specific framework for future research examining the relationship between mindfulness and emotional intelligence.

## 1. Introduction

Teaching requires sustained interaction with students while simultaneously responding to multiple and often competing professional demands that necessitate continuous attention, emotional regulation, and rapid decision-making. Over time, these demands may contribute to increasingly automatized patterns of behavior, reduced conscious awareness of present experiences, chronic stress, and psychological burden (S. Johnson et al., 2005; Hj Ramli et al., 2018; Roeser et al., 2012; Siegel, 2007; West et al., 2001). Nevertheless, teachers are expected to remain calm, sustain attention, regulate their emotions, maintain effective interpersonal relationships, and respond adaptively to complex professional situations (Frank et al., 2016; Roeser et al., 2012; Schutz & Zembylas, 2009; Zapf, 2002). Within this context, mindfulness has received increasing attention as a potentially relevant resource for supporting teachers in managing the psychological and interpersonal demands of their work.

Empirical research provides growing support for the potential relevance of mindfulness in teaching. A 2026 meta-analysis of 13 randomized controlled trials involving 2119 teachers found that mindfulness-based interventions were associated with reductions in perceived stress (SMD = −2.37, 95% CI [−4.40, −0.35]) and negative emotions (SMD = −0.95, 95% CI [−1.27, −0.62]), as well as increased mindfulness (SMD = 0.59, 95% CI [0.39, 0.79]), although substantial methodological and clinical heterogeneity warrants cautious interpretation (Yan et al., 2026). Similarly, a systematic review of 39 studies involving K–12 teachers reported generally favorable effects of mindfulness-based interventions on stress, burnout, and other psychological outcomes, while also identifying considerable heterogeneity in intervention characteristics and outcome measures (Hidajat et al., 2023). Recent intervention research has further reported medium-to-large improvements in teachers’ mindfulness (δ = 0.72), perceived stress (δ = 0.90), and emotional exhaustion (δ = 1.13) (Wagner et al., 2024). Taken together, these findings suggest that mindfulness may be relevant to teachers’ psychological and professional functioning and underscore the importance of examining how mindfulness is conceptualized and measured within the specific context of schools.

Mindfulness is also theoretically relevant to emotional intelligence. Contemporary models of emotional intelligence emphasize the ability to perceive, understand, regulate, and effectively use emotions in oneself and others (Mayer et al., 2016). Although mindfulness and emotional intelligence are distinct constructs, several components of mindfulness, including present-moment awareness, self-observation, emotion regulation, nonjudgmental acceptance, and interpersonal sensitivity, are conceptually relevant to emotionally intelligent functioning (Brown & Ryan, 2003; Schutte & Malouff, 2011). Accordingly, examining mindfulness in educational settings may provide a theoretical basis for future research investigating its relationships with the self-regulatory and interpersonal processes associated with emotional intelligence.

Despite growing attention to mindfulness interventions in educational research (Hwang et al., 2017), questions remain regarding how mindfulness should be defined, operationalized, and measured (Baer et al., 2006; Sauer et al., 2013). Existing measures reflect substantially different conceptualizations of the construct. For example, the Mindful Attention and Awareness Scale (MAAS; Brown & Ryan, 2003) primarily conceptualizes mindfulness in terms of present-moment attention and awareness, whereas the Five Facet Mindfulness Questionnaire (FFMQ; Baer et al., 2006) adopts a multidimensional approach encompassing observing, describing, acting with awareness, nonjudging, and nonreactivity. The Cognitive and Affective Mindfulness Scale–Revised (CAMS-R; Feldman et al., 2007) similarly represents mindfulness through multiple components while treating acceptance and non-reactivity as closely related processes. Despite these different approaches, concerns remain that general mindfulness instruments may rely on relatively abstract or decontextualized items that do not fully capture how mindfulness is experienced within particular populations and settings (Bergomi et al., 2013; Walker, 2011). This limitation has prompted calls for greater attention to the cultural, demographic, and contextual conditions under which mindfulness is conceptualized and measured (Bishop et al., 2004; Burg et al., 2017; Collins et al., 2009; Davidson, 2010; Dobkin, 2008; Frewen et al., 2008; Frewen et al., 2011; Grossman, 2008; Williams, 2010).

These measurement concerns are particularly relevant because mindfulness is increasingly understood as a culturally and contextually embedded construct rather than a universal psychological characteristic (Nilsson & Kazemi, 2016; Oman, 2016, 2018, 2025; Sandage & Stein, 2024). Different theoretical traditions consequently emphasize different dimensions and processes of mindfulness (Baer et al., 2006; Bishop et al., 2004; Brown & Ryan, 2003; Kabat-Zinn, 2003; Nilsson & Kazemi, 2016), while cross-cultural research indicates that its dimensional structure and relationships with psychological adjustment may vary across cultural contexts (Christopher et al., 2009; Ghorbani et al., 2009; Grossman & Van Dam, 2011; Karl et al., 2020; Reyes-Bossio et al., 2022). Mindfulness interventions and measures may therefore require cultural and contextual adaptation because particular dimensions can become more or less salient across cultural, organizational, and occupational settings (Alnabilsy et al., 2025; Baer, 2019; W. W. Li et al., 2024; Paz & Davidovitch, 2025; Sauer et al., 2013; Van Gordon et al., 2015).

This issue is especially relevant to schools, which are complex organizational environments characterized by continuous interpersonal interactions, multiple stakeholder relationships, and institutional demands (Bidwell, 2001; Bossert et al., 1982; Cusick, 1992; Maslowski, 2003). Research suggests that mindfulness within educational settings is multidimensional and influenced by contextual factors that should be considered when developing interventions and measurement instruments (Baelen et al., 2023; Hemming & Hailwood, 2024; Karl et al., 2020; Lee et al., 2024; Renshaw & Phan, 2023). Thus, exploring teachers’ understandings of mindfulness within their professional environments before quantitative operationalization may enhance the contextual relevance and construct validity of school-based measures. Dimensions emerging from such inquiry need not necessarily represent manifestations of a single underlying trait but may instead capture complementary aspects of teachers’ professional experiences.

Qualitative inquiry provides an appropriate foundation for this purpose because it enables researchers to examine mindfulness through teachers’ lived experiences rather than relying exclusively on predefined theoretical assumptions (Albrecht, 2018; Bernay, 2014; Shapiro et al., 2008). Phenomenological and interpretative approaches recognize that mindfulness may be experienced differently across educational and sociocultural contexts and can reveal organizational norms and contextual influences embedded within school life (Baer, 2003; Dunne, 2020; E. J. Langer, 1992; Á. I. Langer et al., 2020; Lorentz, 2011; Lundh, 2020; Molina et al., 2023; Penley, 2018; Styers, 2019; Tonga & Tantekin Erden, 2020). Such approaches can therefore provide an empirical basis for developing psychometrically sound, culturally meaningful, and educationally relevant measurement instruments and interventions (Albrecht, 2018; Oman, 2021).

These qualitative insights must subsequently be operationalized and psychometrically evaluated through quantitative methods. Exploratory sequential mixed methods research is particularly appropriate because it integrates contextual qualitative understanding with quantitative validation, thereby supporting interpretive depth and generalizability (Creswell, 2013; Greene et al., 1989; R. B. Johnson & Onwuegbuzie, 2004; R. B. Johnson et al., 2007; Schoonenboom & Johnson, 2017). Qualitative findings can thus be systematically translated into measurable constructs and subsequently evaluated in larger samples, fulfilling the developmental and complementarity purposes of mixed methods research (Creswell, 2013; Greene et al., 1989; Huynh et al., 2018; R. B. Johnson & Onwuegbuzie, 2004; R. B. Johnson et al., 2007; Kempson, 2012; Phan et al., 2022; Zenner et al., 2014).

The present study examines mindfulness within the Turkish educational context, where relatively bureaucratic and hierarchical organizational structures may shape teachers’ professional experiences and interpretations of mindfulness (Kılınç et al., 2024). Rather than assuming that existing measures fully capture mindfulness as experienced within schools, the study adopts a context-sensitive approach grounded in teachers’ lived experiences. Accordingly, an exploratory sequential mixed methods design was employed to explore teachers’ experiences of mindfulness and subsequently develop and validate the Mindfulness in School Scale (MSS). By integrating phenomenological inquiry with psychometric validation, the study seeks to provide a multidimensional instrument representing distinct yet complementary aspects of teachers’ mindfulness in school settings. The resulting framework may also provide a basis for future research examining theoretical relationships between school-based mindfulness and related constructs, including emotional intelligence.

To achieve this objective, the study addresses the following research questions:

1. How do teachers experience mindfulness in their professional lives?
2. How can the qualitative findings be translated into the development of the Mindfulness in School Scale (MSS)?
3. What evidence supports the content validity and reliability of the dimensions of the Mindfulness in School Scale (MSS)?
4. What is the underlying factor structure of the Mindfulness in School Scale (MSS) as identified through Exploratory Factor Analysis (EFA)?
5. To what extent is the factor structure of the Mindfulness in School Scale (MSS) supported through Confirmatory Factor Analysis (CFA)?

## 2. Materials and Methods

### 2.1. Research Design

This study employed a mixed methods research design incorporating both qualitative and quantitative components to gain a comprehensive understanding of teachers’ experiences of mindfulness and to develop and validate a multidimensional instrument assessing distinct dimensions of teachers’ mindfulness in school settings. Among mixed methods designs, an exploratory sequential design was adopted, in which qualitative data were collected and analyzed first, followed by the development of a quantitative measurement instrument based on the qualitative findings (Creswell, 2013).

The qualitative phase employed a phenomenological research design. Phenomenology seeks to uncover the essence of phenomena that individuals are aware of but have not fully articulated or deeply understood, thereby revealing both explicit and implicit dimensions of lived experience (Creswell, 2013). Accordingly, this phase explored teachers’ lived experiences and conceptualizations of mindfulness within school settings (Lorentz, 2011; Bergomi et al., 2013). Building upon the findings of the qualitative inquiry, the Mindfulness in School Scale (MSS) was developed. The quantitative phase evaluated the psychometric properties of the newly developed MSS. A descriptive survey design was considered appropriate because it is intended to describe existing phenomena as they naturally occur within a population, making it well suited for evaluating the psychometric properties of the newly developed instrument (Creswell, 2013).

### 2.2. Participants

For the qualitative phase of the study, participants were selected using purposive sampling to obtain a comprehensive understanding of mindfulness experiences within school settings. Specifically, maximum variation sampling was employed to capture the broadest possible range of mindfulness experiences among participants. Maximum variation sampling aims to include individuals who differ across characteristics relevant to the phenomenon under investigation, thereby enhancing the diversity and richness of the data (Creswell, 2013). Accordingly, diversity was sought with respect to participants’ years of professional experience, age, gender, educational attainment, and teaching specialization. Recommendations from experts in the field were also considered during the recruitment process to ensure maximum variation within the sample. Data saturation was achieved following semi-structured interviews with 23 teachers.

Participants in the quantitative phase were recruited using convenience sampling from public schools in eastern Türkiye, primarily in the provinces of Kars and Iğdır. Data collection was conducted through in-person visits to schools, during which teachers were invited to participate voluntarily in the study. The EFA and CFA data were collected separately, with the first independent sample (*n* = 397) used for exploratory factor analysis and a subsequently recruited independent sample (*n* = 266) used for confirmatory factor analysis. All participants were employed in public schools. Although only a small number of teachers declined to participate, the exact number of teachers approached was not systematically recorded; therefore, a precise response rate could not be calculated. Similarly, the exact number of participating schools was not retained. Given the use of convenience sampling and the geographical concentration of participants in eastern Türkiye, the quantitative samples should not be considered nationally representative of the broader population of teachers in Türkiye.

The EFA sample (*n* = 397) was predominantly female (71.8%), with male teachers comprising 28.2% of the sample. Approximately 60% of the participants were married, and 87.9% held a bachelor’s degree. The largest age group consisted of teachers aged 29–35 years (40.6%), while the largest professional-experience category comprised teachers with nine or more years of service (37.3%). Regarding school size, participants were drawn from schools of varying enrollment levels, with 28.2% working in schools enrolling 351 or more students.

The independent CFA sample (*n* = 266) showed a broadly similar demographic profile. Women represented 68.4% of the sample and men 31.6%. In terms of marital status, 56.8% were married and 43.2% were single, while 89.5% held a bachelor’s degree. The largest age category was 29–35 years (36.5%), closely followed by teachers aged 22–28 years (35.3%). Regarding professional experience, 41.4% had between one and four years of service, whereas 33.8% had nine or more years of service. Participants most frequently worked in medium-sized schools enrolling between 151 and 350 students.

Comparability analyses were conducted to determine whether the independent EFA and CFA samples differed in their demographic and professional characteristics. Pearson chi-square tests of independence revealed no statistically significant differences between the two samples with respect to age, gender, professional experience, educational attainment, marital status, or school size (all *p* > .05). The distributions of participants across the examined categories were generally similar in both samples, indicating that neither sample was characterized by substantial demographic or professional differences relative to the other. Taken together, these findings suggest that the EFA and CFA samples were broadly comparable across the characteristics examined, thereby supporting the use of the two independent samples in the exploratory and confirmatory stages of the psychometric evaluation of the MSS.

### 2.3. Procedure

Consistent with the exploratory sequential mixed methods design, the study began with a comprehensive review of the literature to inform the development of the qualitative interview protocol. The interview questions were subsequently evaluated by subject-matter experts to establish content relevance and clarity. Following expert review, the interview protocol was pilot-tested with a small group of teachers and minor revisions were made prior to data collection.

Qualitative data were collected through semi-structured interviews and analyzed using content analysis. The resulting themes and codes served as the conceptual foundation for the development of the Mindfulness in School Scale (MSS). In addition to the existing literature, the qualitative findings informed the generation of scale dimensions and item content, ensuring that the instrument reflected teachers’ lived experiences of mindfulness within school settings.

Consistent with the exploratory sequential design, no a priori factor structure or predetermined number of factors was specified for the EFA, allowing the dimensional structure of the MSS to emerge empirically from the data. Following item development and expert review, the preliminary MSS underwent two pilot stages before the main quantitative analyses. An initial comprehensibility pilot was conducted with 14 educators to evaluate item clarity and interpretation, followed by quantitative pilot testing with 96 educators to examine the preliminary functioning of the instrument. The resulting version was subsequently administered to an independent sample for EFA, and the factor structure identified through EFA was evaluated using a separately recruited sample through CFA. The detailed item-development and refinement process is described in the following section.

### 2.4. Questionnaire and Measure

**Figure 1:**
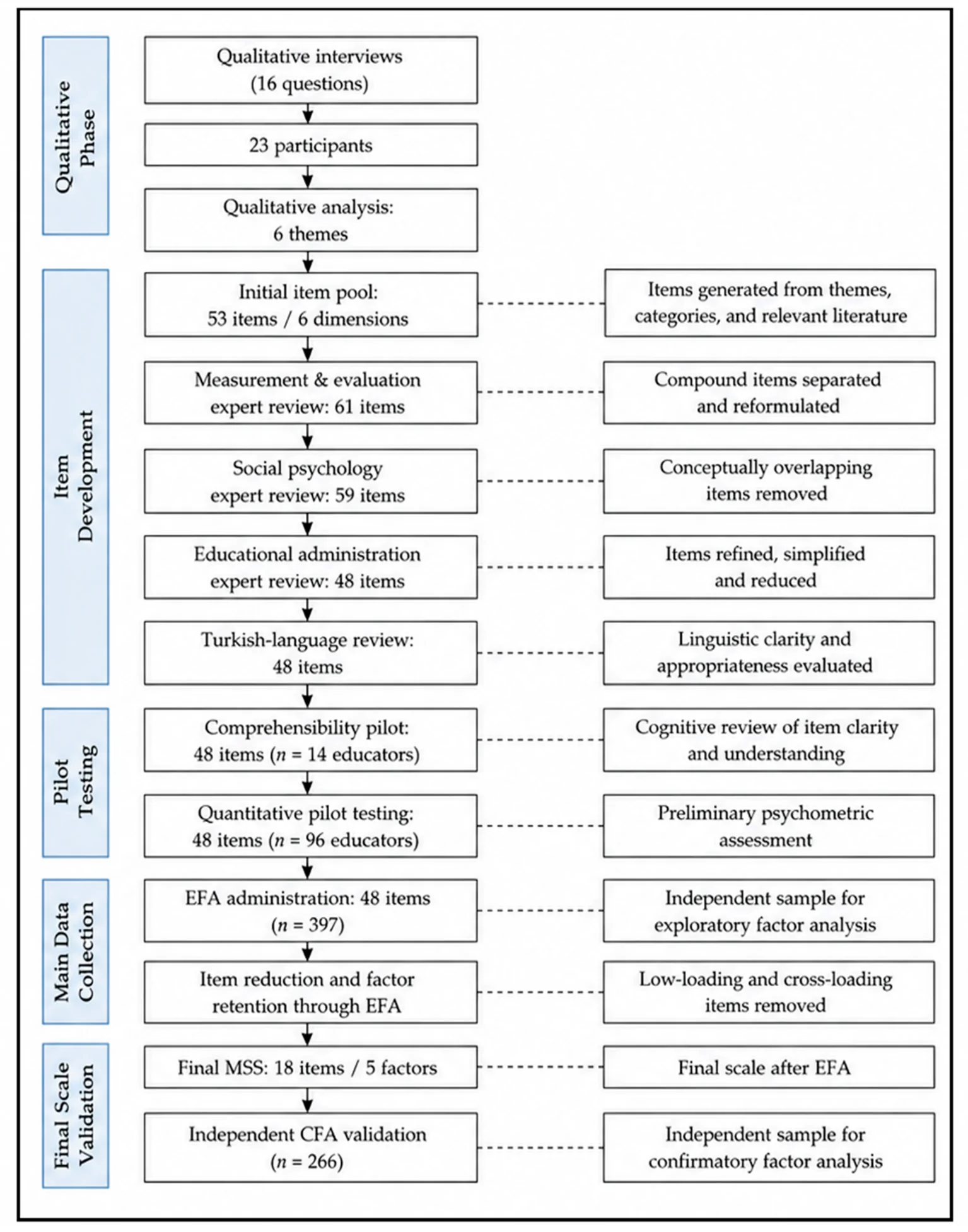
Item Development and Validation Process of the Mindfulness in School Scale (MSS).

The development of the Mindfulness in School Scale (MSS) followed the exploratory sequential mixed methods design adopted in this study as shown in Figure 1. The initial pool of interview questions for the qualitative phase was generated through a comprehensive review of the literature, together with insights from academics and experienced teachers. This process resulted in an initial pool of 19 semi-structured interview questions. The interview protocol was subsequently reviewed by three experts in educational sciences and two experts in educational measurement and evaluation to establish its content validity and clarity. Based on their recommendations, the number of interview questions was reduced to 17. The revised interview protocol was then pilot-tested with four teachers who did not participate in the main study. Following the pilot interviews and participants’ feedback, one additional question was removed. The final semi-structured interview protocol consisted of 16 questions, which were evaluated in terms of content coverage, clarity, and effectiveness before being used for data collection.

Qualitative data obtained through these interviews were analyzed, and the resulting themes and codes served as the conceptual basis for developing the MSS. Rather than relying solely on existing theoretical conceptualizations of mindfulness, the scale items were grounded in teachers’ lived experiences and subsequently refined through evidence from the literature.

Based on the six themes, associated categories, and codes identified during the qualitative phase, together with the relevant mindfulness literature, an initial pool of 53 items representing six preliminary dimensions was generated. The item pool subsequently underwent multiple stages of expert evaluation. Following review by two measurement and evaluation experts, items containing multiple or closely connected statements were separated and reformulated where necessary, increasing the pool from 53 to 61 items. A social psychology expert subsequently evaluated conceptual overlap among the items, resulting in a 59-item version. Four educational administration experts then evaluated the items in terms of conceptual relevance, clarity, and feasibility for use in school settings, resulting in a reduced 48-item version. Finally, two Turkish-language experts evaluated the linguistic clarity and appropriateness of the items, with all 48 items retained.

The resulting 48-item, six-dimensional preliminary instrument first underwent a comprehensibility pilot with 14 teachers to identify potential difficulties in item wording and interpretation. The instrument was subsequently subjected to quantitative pilot testing with a separate sample of 96 teachers before the main scale-development analysis. Following these preliminary stages, the 48-item version was administered to an independent sample of 397 participants for EFA. Item reduction and factor-retention procedures resulted in the final 18-item MSS comprising five factors. The resulting five-factor measurement model was subsequently evaluated through CFA using a separately recruited independent sample of 266 participants. Thus, although the six dimensions derived from the qualitative phase informed the initial item-generation process, they did not predetermine the number of factors ultimately retained through empirical psychometric evaluation.

The Mindfulness in School Scale (MSS) consists of 18 items representing a broad range of professional and emotional experiences encountered by teachers in school settings. The items assess teachers’ capacity for conscious observation of themselves and their surroundings, awareness of internal and external experiences, and nonjudgmental acceptance. Exploratory and confirmatory factor analyses supported a five-factor structure comprising Self-Observation, Environmental Observation, Self-Awareness, Environmental Awareness, and Acceptance.

Self-Observation reflects teachers’ conscious attention to their own language, behaviors, and professional interactions with students, colleagues, and parents. Environmental Observation assesses teachers’ ability to recognize and interpret the emotional climate and needs of students, parents, and other members of the school community. Self-Awareness captures teachers’ awareness of attentional lapses, automatic behaviors, and tendencies to complete professional tasks without full conscious engagement. Environmental Awareness reflects teachers’ awareness of school conditions, institutional expectations, organizational processes, and the needs of educational stakeholders. Finally, Acceptance measures teachers’ ability to acknowledge and manage negative or challenging school experiences without becoming overwhelmed by psychological distress or professional discouragement.

Although Reactive Control was represented in the initial six-dimensional item pool, it did not emerge as an empirically distinct dimension during the quantitative scale-development process and showed insufficient differentiation from Acceptance. Consequently, Reactive Control was not retained as a separate factor in the final MSS. The empirical basis for this decision is reported in the EFA results, while its theoretical implications are considered further in the Discussion. Collectively, these dimensions reflect contemporary multidimensional conceptualizations of mindfulness while remaining firmly grounded in the authentic experiences of teachers working in school settings.

The MSS employs a five-point Likert-type response format to assess teachers’ mindfulness in school contexts. Negatively worded items representing reduced awareness or difficulties in acceptance (Items 8, 9, 10, 11, 16, 17, and 18) are reverse scored. Higher scores on each dimension indicate higher levels of that aspect of mindfulness, whereas lower scores indicate lower levels of that aspect of mindfulness.

The psychometric properties of the MSS were evaluated through both Exploratory Factor Analysis (EFA) and Confirmatory Factor Analysis (CFA). The five-factor solution explained a substantial proportion of the total variance (Factor 1 = 22.54%, Factor 2 = 7.04%, Factor 3 = 5.64%, Factor 4 = 4.32%, and Factor 5 = 11.92%). The instrument also demonstrated satisfactory internal consistency. Cronbach’s alpha coefficients for the five subscales ranged from 0.80 to 0.93 (Self-Observation, α = 0.93; Environmental Observation, α = 0.80; Self-Awareness, α = 0.84; Environmental Awareness, α = 0.86; and Acceptance, α = 0.83). Furthermore, corrected item–total correlations provided evidence for the internal consistency of the respective dimensions.

### 2.5. Data Analysis

#### 2.5.1. Qualitative Data Analysis

Phenomenological research requires systematic and rigorous analytical procedures to uncover the essence of participants’ lived experiences (Patton, 2014). Accordingly, the qualitative data collected through the semi-structured interviews were analyzed using content analysis, a method that facilitates the systematic reduction, organization, and interpretation of qualitative data to identify meaningful patterns and themes (Patton, 2014).

The analysis combined inductive and deductive approaches. Consistent with Corbin and Strauss (2014), the analytical process began with an inductive approach through open coding, allowing themes, categories, and patterns to emerge directly from the participants’ narratives rather than being imposed a priori. An initial codebook was developed from the interview transcripts, and similar codes were subsequently grouped into broader categories and overarching themes. Following the inductive coding process, a deductive analysis was conducted to examine the conceptual consistency of the emergent codes, categories, and themes with the existing mindfulness literature. The entire coding framework was repeatedly reviewed and refined by comparing the qualitative findings with relevant theoretical perspectives. Codes, categories, and themes that were inconsistent with the literature or conceptually ambiguous were critically re-evaluated and revised where necessary. Thus, while the generation of codes and themes was primarily data-driven, their refinement and interpretation were guided by existing theoretical and empirical evidence.

To enhance the methodological rigor of the qualitative phase, multiple strategies were employed to establish trustworthiness, including credibility, transferability, dependability, and confirmability (Lincoln & Guba, 1985). Credibility was strengthened through data triangulation by collecting perspectives from different teacher groups. Theoretical triangulation was achieved by interpreting the findings through multiple conceptualizations of mindfulness. In addition, member checking was conducted by sharing the coded transcripts and thematic interpretations with a participant to verify that the findings accurately represented their experiences. Data collection continued until saturation was achieved, and the analytical process was continuously reviewed through peer debriefing with the thesis supervisory committee.

Transferability was enhanced by providing rich descriptions of the research context, participant characteristics, and analytical procedures, supported by illustrative quotations from participants. Rather than seeking statistical generalizability, the study aimed to achieve analytical transferability, enabling readers to determine the applicability of the findings to comparable educational contexts.

Dependability was established through the maintenance of a comprehensive audit trail documenting each stage of the research process, from data collection and coding to analysis and reporting. To evaluate coding consistency, an independent qualitative researcher coded the interview data, resulting in an inter-coder agreement of 88%, indicating high coding reliability (Miles & Huberman, 1994).

Confirmability was addressed through continuous reflexive practice. Throughout the study, the researcher critically reflected on personal assumptions, disciplinary background, and potential sources of bias that could influence data interpretation. Recognizing that prior expertise in emotional intelligence and familiarity with mindfulness research might shape analytical judgments, the researcher adopted a reflexive and neutral stance during both data collection and analysis. Particular care was taken to avoid leading questions or researcher-driven interpretations, thereby ensuring that the findings remained grounded in participants’ lived experiences rather than the researcher’s preconceptions.

#### 2.5.2. Quantitative Data Analysis

To evaluate the psychometric properties of the Mindfulness in School Scale (MSS), a series of statistical analyses was conducted in IBM SPSS (25) and AMOS (23). Prior to factor analysis, the distributional properties of the EFA sample were examined using skewness and kurtosis statistics, and missing data were handled using listwise deletion. Skewness values ranged from −1.073 to 0.047, whereas kurtosis values ranged from −0.576 to 1.263, indicating no severe departures from univariate normality. Although the MSS items were assessed using a five-point Likert response format, they were treated as approximately continuous for factor-analytic purposes. Simulation research suggests that treating ordinal variables as continuous becomes more defensible when five or more response categories are available, although estimator performance also depends on distributional characteristics, sample size, and category thresholds (Rhemtulla et al., 2012). Accordingly, conventional Maximum Likelihood (ML) estimation was used for both the EFA and CFA. No robust or weighted least-squares estimator (e.g., MLR, WLSMV, or DWLS) was employed. The use of conventional ML was considered appropriate given the five response categories, the observed distributional characteristics, and the sample sizes (C.-H. Li, 2016; Rhemtulla et al., 2012).

The construct validity of the MSS was initially examined through Exploratory Factor Analysis (EFA). Prior to factor extraction, the suitability of the data for factor analysis was evaluated using the Kaiser–Meyer–Olkin (KMO) measure of sampling adequacy and Bartlett’s Test of Sphericity. The KMO coefficient was 0.821, and Bartlett’s Test of Sphericity was statistically significant, χ^2^(153) = 2471.74, *p* < .001, supporting the suitability of the correlation matrix for factor analysis. EFA was conducted using Maximum Likelihood extraction with Direct Oblimin rotation because the underlying dimensions of mindfulness were theoretically expected to be correlated (Tabachnick & Fidell, 2019). The number of factors to retain was determined using parallel analysis, in which eigenvalues obtained from the observed data were compared with those generated from simulated random data. During factor extraction, item retention was based on both statistical and conceptual considerations. Although a minimum factor loading of 0.32 is generally considered acceptable (Tabachnick & Fidell, 2019), items with loadings below approximately 0.40 or exhibiting substantial cross-loadings were removed to improve the interpretability and psychometric quality of the factor solution (Howard & Henderson, 2023).

The factor structure identified through EFA was subsequently evaluated using Confirmatory Factor Analysis (CFA) with an independent sample. Standardized CFA factor loadings ranged from 0.50 to 0.90. Model fit was assessed using multiple indices, including the chi-square statistic (χ^2^), degrees of freedom (df), χ^2^/df ratio, Standardized Root Mean Square Residual (SRMR), Root Mean Square Error of Approximation (RMSEA) with its 90% confidence interval, Goodness-of-Fit Index (GFI), Adjusted Goodness-of-Fit Index (AGFI), Normed Fit Index (NFI), Tucker–Lewis Index (TLI), and Comparative Fit Index (CFI). In addition to the proposed correlated five-factor model, one-factor and bifactor models were estimated to examine alternative representations of the MSS structure. Competing models were compared using overall fit indices and information criteria, including the Akaike Information Criterion (AIC) and Bayesian Information Criterion (BIC).

Reliability was evaluated using Cronbach’s alpha, McDonald’s omega, composite reliability (CR), corrected item–total correlations, and split-half reliability. Convergent validity was examined using Average Variance Extracted (AVE), whereas discriminant validity was assessed using the Fornell–Larcker criterion and the heterotrait–monotrait ratio (HTMT). Potential common method variance was examined using Harman’s single-factor test. Finally, measurement invariance across gender was assessed using sequential multi-group CFA, including configural, metric, and scalar invariance models. Changes in CFI and RMSEA across successive models were used to evaluate measurement invariance. A ready-to-use version of the MSS, including the response format, subscale composition, and scoring instructions, is provided in the Supplementary Materials.

## 3. Results

Consistent with the exploratory sequential mixed methods design, the findings are presented in two stages. First, the qualitative findings are reported to provide an in-depth understanding of participants’ experiences and conceptualizations of mindfulness. These findings served as the foundation for the development of the Mindfulness in School Scale (MSS). Subsequently, the quantitative findings are presented, including the psychometric evaluation of the MSS and the results of the statistical analyses.

### 3.1. Qualitative Results

The qualitative analysis identified six overarching themes and thirteen categories that captured teachers’ experiences and conceptualizations of mindfulness within school settings. These themes and categories emerged from semi-structured interviews conducted with 23 teachers and provide an in-depth understanding of how mindfulness is experienced and interpreted in everyday school life.

To illustrate each category, representative quotations that best reflected participants’ experiences were selected and are presented throughout the findings. These quotations provide insight into the ways in which teachers perceive and enact mindfulness within their professional contexts. Table 1 presents an overview of the themes and categories that emerged from the qualitative analysis.

As shown in Table 1, the qualitative analysis yielded six overarching themes and thirteen categories that characterize teachers’ experiences of mindfulness in school settings. To facilitate the interpretation of these findings, representative participant quotations are presented for each category. These quotations were selected because they succinctly capture the essence of each category and provide direct evidence supporting the thematic interpretations. Presenting representative quotations alongside the themes and categories enhances the transparency and credibility of the qualitative findings.

The phenomenological analysis identified six major themes and thirteen categories reflecting participants’ experiences of mindfulness in school settings. The first theme, Self-Observation, encompassed the categories of Behavioral Observation and Emotional Observation. Behavioral Observation reflected participants’ conscious monitoring of their professional behaviors and interactions within the formal and rule-governed nature of school environments, whereas Emotional Observation captured their awareness of their own emotional experiences throughout the school day. Together, these findings suggest that teachers engage in continuous self-monitoring while performing their professional roles.

The second theme, Environmental Observation, comprised the categories of Empathy and Observation of the School Environment. Participants described mindfulness as involving both an empathic understanding of students and other school stakeholders and an active awareness of the broader school environment, including its physical conditions, interpersonal climate, and the changing characteristics of students and parents. Collectively, the findings from the first two themes indicate that mindfulness in schools involves simultaneous awareness of both internal experiences and the external educational environment.

One of the most salient findings of the study was the theme of Self-Awareness, which included the categories of Self-Awareness Experiences, Perceived Effects of Self-Awareness, and Experiences Hindering Self-Awareness. Participants described self-awareness as facilitating concentration, motivation, and constructive problem-solving, particularly in challenging professional situations. At the same time, excessive workload and time pressure emerged as major organizational barriers that reduced teachers’ capacity to remain mindful during their daily professional activities. These findings suggest that mindfulness extends beyond an internal psychological process and has important implications for teachers’ professional functioning and their capacity to respond effectively to workplace challenges.

Similarly, the theme of Environmental Awareness consisted of two categories: Awareness of School Stakeholders and Professional Awareness. Participants demonstrated an awareness of students’ individual potential while simultaneously recognizing the responsibilities and expectations associated with their professional roles. These findings illustrate that mindfulness is closely connected to teachers’ professional commitment and their ability to respond thoughtfully to the needs of the school community.

Finally, the themes of Acceptance and Reactive Control reflected how mindfulness was translated into behavioral responses within school settings. The categories of Acceptance and Non-Acceptance illustrated participants’ efforts either to acknowledge and learn from mistakes or to struggle with persistent negative thoughts and emotional resistance. Likewise, the categories of Controlling Reactions and Expressing Reactions captured two contrasting approaches to managing interpersonal conflict: participants described either intentionally regulating their emotional responses to de-escalate conflict or reacting impulsively through expressions of anger. Taken together, these findings suggest that mindfulness in school settings is a multidimensional framework comprising both intrapersonal dimensions (e.g., self-observation, self-awareness, and acceptance) and interpersonal or contextual dimensions (e.g., environmental observation, environmental awareness, and reactive control). Moreover, participants’ accounts indicate that organizational factors, particularly excessive workload, may substantially influence the extent to which mindfulness can be maintained in everyday educational practice.

### 3.2. Quantitative Results

To examine the construct validity of the Mindfulness in School Scale (MSS), an Exploratory Factor Analysis (EFA) was conducted using the Maximum Likelihood extraction method. Because the underlying dimensions of mindfulness were theoretically expected to be correlated, Direct Oblimin rotation was employed (Tabachnick & Fidell, 2019). To obtain a parsimonious and theoretically meaningful factor solution, a relatively stringent item-retention criterion was adopted. Although a minimum factor loading of 0.32 is generally considered acceptable (Tabachnick & Fidell, 2019), items with factor loadings below 0.40 or exhibiting substantial cross-loadings were removed to improve the interpretability and psychometric quality of the scale (Howard & Henderson, 2023).

The number of factors to retain was determined using parallel analysis. Parallel analysis supported the retention of five factors, as the real-data component eigenvalues for the first five factors (4.584, 2.697, 1.711, 1.376, and 1.175) exceeded their corresponding simulated mean eigenvalues (1.393, 1.312, 1.259, 1.211, and 1.163). In contrast, the sixth real-data eigenvalue (0.754) was lower than the corresponding simulated mean eigenvalue (1.125), indicating that an additional factor should not be retained. Accordingly, five factors were retained. Following Maximum Likelihood extraction, the five-factor solution accounted for 51.46% of the total variance.

Although the qualitative phase identified six themes, including Reactive Control, the EFA supported a five-factor rather than a six-factor structure. Items corresponding to Reactive Control had been included in the initial item pool; however, Reactive Control did not emerge as an empirically distinct factor during the factor-analytic process. Instead, the findings indicated insufficient differentiation between Reactive Control and Acceptance to justify retaining them as separate dimensions. Accordingly, Reactive Control was not retained as an independent factor in the final MSS. This resulted in a five-factor structure comprising Self-Observation, Environmental Observation, Self-Awareness, Environmental Awareness, and Acceptance.

Following this refinement process, the final version of the Mindfulness in School Scale (MSS) consisted of 18 items distributed across five factors. The factor structure, standardized factor loadings obtained from both the EFA and CFA, and the proportion of variance explained by each factor are presented in Table 2.

As shown in Table 2, the five factors were identified as Self-Observation, Environmental Observation, Self-Awareness, Environmental Awareness, and Acceptance. The EFA factor loadings ranged from 0.51 to 0.93, indicating that all retained items demonstrated satisfactory associations with their respective latent factors. To validate the factor structure identified through the EFA, a Confirmatory Factor Analysis (CFA) was subsequently performed. The results indicated that all 18 items significantly loaded on their hypothesized latent constructs (*p* < .05), with standardized factor loadings ranging from 0.50 to 0.90. The five-factor structure of the MSS explained 51.46% of the total variance. These findings provide evidence that each item adequately represents its intended construct.

To further evaluate the construct validity of the MSS, the goodness-of-fit of the five-factor measurement model was assessed using CFA. The model fit was evaluated using multiple fit indices, including the chi-square to degrees of freedom ratio (χ^2^/df), the Root Mean Square Error of Approximation (RMSEA), the Goodness-of-Fit Index (GFI), the Adjusted Goodness-of-Fit Index (AGFI), the Normed Fit Index (NFI), the Tucker–Lewis Index (TLI), and the Comparative Fit Index (CFI). The model fit indices are presented in Table 3.

The five-factor structure identified through the EFA was subsequently evaluated using Confirmatory Factor Analysis (CFA). All standardized factor loadings were statistically significant (*p* < .05) and ranged from 0.50 to 0.90, indicating that each item adequately represented its intended latent construct. Inspection of the modification indices suggested that no theoretically justifiable model modifications were required. The measurement model demonstrated a satisfactory to good overall fit to the data, χ^2^(125) = 150.331, *p* = .061, χ^2^/df = 1.20, SRMR = 0.047, RMSEA = 0.028, 90% CI [0.000, 0.043], GFI = 0.944, AGFI = 0.923, NFI = 0.914, TLI = 0.981, and CFI = 0.984.

To further evaluate the dimensional structure of the MSS, the correlated five-factor model was compared with one-factor and bifactor alternatives. The one-factor model demonstrated poor fit, χ^2^(135) = 983.854, χ^2^/df = 7.288, CFI = 0.470, TLI = 0.399, RMSEA = 0.154, 90% CI [0.145, 0.163], AIC = 1055.854, and BIC = 1184.859. Although the bifactor model demonstrated acceptable fit, χ^2^(117) = 171.247, χ^2^/df = 1.464, CFI = 0.966, TLI = 0.956, RMSEA = 0.042, 90% CI [0.027, 0.055], AIC = 279.247, and BIC = 472.756, it did not outperform the correlated five-factor model. The correlated five-factor model showed better fit, χ^2^(125) = 150.331, χ^2^/df = 1.203, CFI = 0.984, TLI = 0.981, RMSEA = 0.028, 90% CI [0.000, 0.043], AIC = 242.301, and BIC = 407.141. In addition, the standardized loadings of the three Acceptance items on the general factor in the bifactor model were negligible (−0.135, −0.023, and 0.022), whereas their loadings on the specific Acceptance factor remained substantial (0.565, 0.932, and 0.761). Overall, these findings support the correlated five-factor representation of the MSS over the one-factor and bifactor alternatives.

To evaluate the reliability of Mindfulness in School Scale (MSS), Cronbach’s alpha coefficients, corrected item-total correlations, and split-half reliability coefficients were calculated. The reliability estimates for each subscale are presented in Table 4.

As shown in Table 4, corrected item–total correlations ranged from 0.446 to 0.793, exceeding the recommended minimum value of 0.30 and indicating satisfactory item discrimination. Across the five dimensions, Cronbach’s alpha coefficients ranged from 0.80 to 0.93, McDonald’s omega coefficients ranged from 0.83 to 0.94, and Composite Reliability (CR) values ranged from 0.704 to 0.832, indicating satisfactory to good reliability.

Descriptive statistics and Pearson correlation coefficients were computed to examine the distributional characteristics of the MSS subscales and the relationships among them. The results are presented in Table 5.

As shown in Table 5, the Self-Observation subscale had the highest mean score (*M* = 4.73, *SD* = 0.40), whereas Acceptance had the lowest (*M* = 2.75, *SD* = 0.88). Skewness and kurtosis values indicated no substantial deviations from normality.

The correlation analysis revealed significant positive associations among most MSS dimensions. Self-Observation was moderately correlated with Environmental Observation (*r* = 0.30, *p* < .01) and Environmental Awareness (*r* = 0.44, *p* < .01), and weakly correlated with Self-Awareness (*r* = 0.18, *p* < .01). Environmental Observation was weakly associated with Self-Awareness (*r* = 0.11, *p* < .01) and moderately associated with Environmental Awareness (*r* = 0.41, *p* < .01).

Unlike the other subscales, Acceptance demonstrated a distinct correlation pattern. It was positively associated with Self-Awareness (*r* = 0.31, *p* < .01) but negatively associated with Environmental Awareness (*r* = −0.14, *p* < .01), while showing no significant relationships with the observation-related dimensions. This pattern indicates that Acceptance may function relatively independently from some observation-related dimensions of the MSS.

To further examine the construct validity of the MSS, convergent and discriminant validity were assessed using Average Variance Extracted (AVE) and the Fornell–Larcker criterion. AVE was calculated from the standardized CFA factor loadings. Discriminant validity was evaluated by comparing the square root of the AVE (√AVE) for each dimension with its correlations with the other dimensions. According to the Fornell–Larcker criterion, discriminant validity is supported when the √AVE for each construct exceeds its correlations with the other constructs.

As shown in Table 6, AVE values ranged from 0.394 to 0.622. Self-Observation, Self-Awareness, and Acceptance exceeded the conventional 0.50 criterion, whereas Environmental Observation and Environmental Awareness yielded AVE values of 0.394 and 0.465, respectively, indicating comparatively weaker convergent validity for these two dimensions. Nevertheless, the Fornell–Larcker criterion provided clear evidence of discriminant validity. For each MSS dimension, the square root of AVE exceeded all corresponding inter-factor correlations. The highest observed correlation was 0.44, between Self-Observation and Environmental Awareness, which remained below the relevant √AVE values of 0.789 and 0.682. Thus, the results indicate that the five MSS dimensions are empirically distinguishable from one another, supporting the discriminant validity of the five-factor measurement model. Discriminant validity was further examined using the heterotrait–monotrait ratio (HTMT). HTMT values ranged from 0.030 to 0.515, with all values remaining below the conservative threshold of 0.85, providing additional evidence for discriminant validity among the five MSS dimensions. Given the relatively low correlations among several dimensions, however, the MSS is interpreted as a multidimensional instrument at the subscale level rather than as a single higher-order mindfulness construct.

Potential common method variance was assessed using Harman’s single-factor test. An unrotated single-factor solution accounted for 20.63% of the total variance, substantially below the commonly used 50% threshold. Thus, the results did not indicate that a single common method factor dominated the variance in the MSS items.

To examine whether the five-factor structure of the MSS operated equivalently across gender, measurement invariance was assessed using sequential multi-group CFA. Configural, metric, and scalar invariance models were tested, with changes in CFI and RMSEA used to evaluate invariance across successive models. The results are presented in Table 7.

Measurement invariance across gender was examined using sequential multi-group CFA. As shown in Table 7, the configural model demonstrated satisfactory fit, χ^2^(250) = 294.799, CFI = 0.973, TLI = 0.967, RMSEA = 0.037, indicating that the five-factor structure was supported across gender groups. Metric invariance was subsequently supported, as constraining factor loadings to equality resulted in only a negligible change in model fit (ΔCFI = −0.004, ΔRMSEA = 0.001). Scalar invariance was also supported after additionally constraining item intercepts to equality (ΔCFI = −0.004, ΔRMSEA = 0.002). Thus, the results supported configural, metric, and scalar measurement invariance of the MSS across gender.

## 4. Discussion

### 4.1. Qualitative Discussion

The qualitative phase of the present study was designed to provide an in-depth understanding of mindfulness as experienced within school settings. Accordingly, the discussion focuses on how teachers conceptualized and experienced mindfulness in their professional contexts. The phenomenological analysis identified six interrelated themes: Self-Observation, Environmental Observation, Self-Awareness, Environmental Awareness, Acceptance, and Reactive Control.

The first two themes, Self-Observation and Environmental Observation, highlight the importance of both intrapersonal and interpersonal awareness in teachers’ conceptualizations of mindfulness. Self-Observation, which reflects individuals’ awareness of their own thoughts, emotions, and behaviors, has been widely recognized as a foundational component of mindfulness (Bergomi et al., 2013; Schussler et al., 2016). In the present study, participants described self-observation primarily through their conscious monitoring of emotions and professional behaviors within the school environment. This finding is consistent with previous research suggesting that mindful self-observation contributes to the development of positive emotions and supports a healthy school climate (Berson & Oreg, 2016; Kang & Whittingham, 2010).

Similarly, the theme of Environmental Observation emphasized participants’ awareness of the emotions, needs, and perspectives of students, colleagues, and parents. Teachers frequently associated mindfulness with empathic understanding, careful attention to others, and responsive leadership behaviors. These findings support previous studies highlighting interpersonal mindfulness as a key component of effective educational leadership and teacher practice (Frank et al., 2016). In particular, participants’ emphasis on empathy and compassion toward students is consistent with earlier research demonstrating that mindfulness promotes empathic relationships and compassionate interactions within educational settings (Arendt et al., 2019; Emerson et al., 2017; Glomb et al., 2011; Jennings et al., 2011; Schussler et al., 2016; Seitz & Angel, 2020).

The theme of Self-Awareness further demonstrates that mindfulness extends beyond momentary attention to include broader professional competencies. Participants associated mindfulness with maintaining attentional focus, self-compassion, self-efficacy, and professional commitment, findings that closely parallel previous qualitative research conducted in educational settings (Bernay, 2014; Burrows, 2015; Flook et al., 2013; Glomb et al., 2011; Gold et al., 2010). Furthermore, participants reported that self-awareness contributed to creating a positive school climate, enhancing psychological well-being, reducing occupational stress, and improving problem-solving and leadership practices. These findings are consistent with a growing body of evidence demonstrating the positive influence of mindfulness on teachers’ well-being, leadership effectiveness, and school functioning (Chiesa & Serretti, 2010; Jennings & Greenberg, 2009; Klocko & Wells, 2015; Mahfouz, 2018; Özgün, 2018).

At the same time, participants identified excessive workload, administrative responsibilities, and psychological strain as significant barriers to maintaining mindfulness. These organizational demands were described as contributing to attentional lapses, reduced concentration, and automatic patterns of behavior, thereby limiting participants’ ability to remain fully aware during their professional activities. This finding is consistent with previous studies identifying occupational stress and workload as important contextual factors that undermine mindfulness among teachers (Frank et al., 2016; Jennings et al., 2013; Kabat-Zinn, 2017; Nilsson & Kazemi, 2016). Taken together, these findings suggest that mindfulness should not be viewed solely as an individual psychological characteristic but also as a context-dependent framework shaped by organizational conditions within schools.

The Environmental Awareness theme further extends the understanding of mindfulness by emphasizing teachers’ awareness of the broader organizational context in which they work. Participants described being aware of students’ capacities, teachers’ expectations, school conditions, and institutional responsibilities. These findings suggest that mindfulness in school settings involves not only awareness of internal experiences but also conscious attention to external organizational conditions. This interpretation is consistent with the view that mindfulness encompasses awareness of both internal and external experiences in the present moment (Dane, 2011). Because schools operate as open systems characterized by continuous interactions with students, colleagues, parents, and the wider community (Bidwell, 2001; Bossert et al., 1982; Cusick, 1992; Maslowski, 2003), teachers’ awareness of these contextual influences represents an important aspect of mindfulness. Previous research has likewise shown that mindfulness enhances sensitivity to others’ perspectives and supports more authentic and responsive interpersonal relationships (Frank et al., 2016; Nilsson & Kazemi, 2016; Reitz et al., 2020; Rupprecht et al., 2019). Accordingly, the present findings suggest that environmental awareness reflects an organizational form of mindful attention that enables teachers to interpret contextual cues and respond more thoughtfully within the dynamic environment of schools.

The final themes, Acceptance and Reactive Control, revealed findings that both converged with and extended the existing mindfulness literature. Participants described acceptance as acknowledging mistakes without excessive self-judgment and viewing challenging experiences as opportunities for learning and professional growth, consistent with established conceptualizations of mindfulness (Bishop et al., 2004; Wells, 2016). However, participants also reported that persistent workload and occupational stress often hindered acceptance, resulting in rumination, negative evaluations, regret, and emotional exhaustion. These findings are consistent with previous research demonstrating that chronic occupational demands may weaken acceptance and contribute to teacher burnout (Jennings et al., 2013).

With respect to Reactive Control, participants frequently described intentionally regulating their immediate emotional reactions and adopting preventive strategies to de-escalate interpersonal conflict, findings that are consistent with previous mindfulness research (Jennings et al., 2013; E. J. Langer, 2000). However, one noteworthy finding was that several participants also viewed immediate emotional expression as a legitimate strategy for resolving workplace conflicts. This perspective contrasts with the predominant assumptions of mindfulness theory, which generally emphasizes non-reactivity and deliberate emotional regulation (Bernay, 2014; Burrows, 2011). This divergence may reflect the influence of cultural and organizational characteristics of Turkish schools, suggesting that the expression and regulation of mindful behavior may vary across sociocultural and institutional contexts (Forbes, 2016; Lau, 2016; Titmuss, 2016). Consequently, these findings raise the possibility that the expression of mindfulness may differ across educational and cultural settings, a hypothesis that requires direct cross-cultural investigation.

### 4.2. Quantitative Discussion

The development of Mindfulness in School Scale (MSS) was motivated by ongoing conceptual and methodological concerns regarding the assessment of mindfulness. Previous studies have criticized existing mindfulness measures for relying heavily on abstract and decontextualized items that may not adequately capture individuals’ lived experiences (Bergomi et al., 2013; Bishop et al., 2004; Brown & Ryan, 2003; Collins et al., 2009; Davidson, 2010; Dobkin, 2008; Frewen et al., 2008; Williams, 2010). Consequently, researchers have recommended developing mindfulness instruments that incorporate the experiences of individuals from different age groups, cultures, genders, and professional backgrounds. Similarly, several authors have argued that mindfulness measures should be operationalized using constructs derived from qualitative inquiry rather than relying exclusively on predefined theoretical models (Grossman, 2008; Frewen et al., 2011). Consistent with these recommendations, the MSS was developed using an exploratory sequential mixed methods design in which the qualitative findings directly informed the generation of scale dimensions and items.

Mindfulness is widely recognized as a culturally and contextually embedded construct, and consequently has been conceptualized differently across theoretical traditions (Nilsson & Kazemi, 2016). Likewise, schools represent complex organizational environments that are continually shaped by interpersonal interactions and contextual influences (Bidwell, 2001; Bossert et al., 1982; Cusick, 1992; Maslowski, 2003). Taken together, these considerations suggest that the assessment of mindfulness in educational settings requires measurement instruments that are sensitive to both cultural and organizational contexts. Failure to consider these contextual influences may limit the validity of mindfulness assessments in school settings and other specialized populations (Bergomi et al., 2013; Grossman, 2008; Frewen et al., 2011). Recent studies have similarly emphasized that mindfulness is a multidimensional phenomenon whose manifestation within schools is influenced by numerous contextual variables, highlighting the importance of developing context-specific measurement instruments (Baelen et al., 2023; Renshaw & Phan, 2023).

Consistent with this context-dependent perspective, the factor structure of the MSS diverges in several respects from existing mindfulness measures. Compared with existing mindfulness measures, the MSS does not include a Describe dimension. With the exception of the Kentucky Inventory of Mindfulness Skills (KIMS) and the Five Facet Mindfulness Questionnaire (FFMQ), the Describe dimension is generally absent from contemporary mindfulness instruments. Moreover, because the FFMQ was developed by integrating items from previously existing scales, its Describe dimension was largely derived from the KIMS (Baer et al., 2004). The KIMS itself was developed within the framework of Dialectical Behavior Therapy. In the present study, the Describe dimension was intentionally excluded because the qualitative findings indicated that mindfulness in school settings is more closely related to teachers’ lived experiences than to their ability to verbally describe internal experiences, consistent with Baer et al. (2006). Furthermore, previous psychometric research has questioned the discriminant validity of the Describe dimension, demonstrating that it often fails to distinguish effectively between individuals with different mindfulness profiles (Josefsson et al., 2011). The absence of this dimension in the MSS therefore appears to be theoretically and empirically justified.

The qualitative findings initially suggested that Reactive Control represented a distinct dimension of mindfulness in school settings, and corresponding items were included during the initial stages of scale development. However, the EFA did not support Reactive Control as an independent factor. This finding is consistent with previous psychometric research demonstrating considerable conceptual overlap between reactive control and acceptance (Baer et al., 2008). Several mindfulness instruments, including the Five Facet Mindfulness Questionnaire (FFMQ), the Southampton Mindfulness Questionnaire (SMQ), and the Freiburg Mindfulness Inventory (FMI), assess aspects of non-reactivity or reactive control (Baer et al., 2008). Nevertheless, other instruments, such as the Cognitive and Affective Mindfulness Scale–Revised (CAMS-R), do not distinguish reactive control from acceptance, instead conceptualizing them as closely related components of a broader acceptance construct (Feldman et al., 2007). Accordingly, the present findings suggest that reactive control may not represent an empirically distinct dimension within school-based mindfulness, but rather an aspect of acceptance, a conclusion that is consistent with previous conceptualizations of mindfulness (Baer et al., 2008; Kalill et al., 2014).

The pattern of relationships among the MSS subscales was broadly consistent with previous multidimensional conceptualizations of mindfulness. Most dimensions demonstrated low to moderate positive correlations, indicating that they represent related yet distinguishable aspects of mindfulness. Similar correlation patterns have been reported during the adaptation of the FFMQ and other mindfulness measures across different cultural contexts (Baer, 2019; Burzler et al., 2019; Giovannini et al., 2014; Noda et al., 2022). Likewise, previous validation studies have consistently shown that the relationships among mindfulness dimensions tend to be modest rather than uniformly strong, supporting the multidimensional nature of mindfulness (Baer et al., 2006; Cebolla et al., 2012; Van Dam et al., 2012).

One particularly noteworthy finding concerns the pattern of correlations involving the Acceptance dimension. Whereas most MSS dimensions were positively associated with one another, Acceptance demonstrated weak or negative relationships with several observation-related dimensions. Similar findings have been reported previously, where the coexistence of high observational ability and high acceptance has proven difficult to establish psychometrically (Lilja et al., 2013). Recent research has suggested that mindfulness may be characterized by distinct profiles, such as accepting mindfulness and judgmental observers, rather than representing a single homogeneous pattern. For example, individuals characterized as judgmental observers may exhibit relatively strong observational tendencies alongside lower levels of acceptance (Zhu et al., 2020). Although this perspective provides one possible theoretical explanation for the weak or negative correlations involving Acceptance in the present study, mindfulness profiles were not empirically examined, and this interpretation therefore remains tentative. Future research should use latent profile analysis to investigate whether distinct mindfulness profiles can be identified among teachers and whether such profiles help explain the observed relationships between Acceptance and the observation-related dimensions. Previous studies have nevertheless emphasized the importance of acceptance in distinguishing between different patterns of mindful functioning (Cebolla et al., 2012; Josefsson et al., 2011; Lilja et al., 2011, 2013).

Another finding requiring consideration is the relatively high mean score for Self-Observation (M = 4.73, SD = 0.40), together with its negative skewness (−0.97), which suggests a potential ceiling effect. This pattern may partly reflect the context-specific nature of the Self-Observation items, which emphasize teachers’ attention to their own behavior during interactions with students, colleagues, and parents. Such responses may capture not only mindful self-observation but also socially desirable responding or conformity to professional expectations. Accordingly, Self-Observation should be interpreted cautiously as a context-specific dimension reflecting attention to one’s behavior within school-based interactions, rather than as a direct representation of the broader intentional and non-judgmental awareness emphasized in general conceptualizations of mindfulness. Future validation studies should examine this possibility by investigating associations between Self-Observation and established measures of social desirability, such as the Marlowe–Crowne Social Desirability Scale.

The construct validity of the MSS was further supported through Confirmatory Factor Analysis, providing additional evidence for the structural validity of the proposed five-dimensional measurement model. The proposed five-factor model demonstrated good model fit across most goodness-of-fit indices. Specifically, the RMSEA, AGFI, CFI, and NFI values indicated satisfactory to good fit, whereas the GFI suggested good model fit. Collectively, these findings provide evidence supporting the structural validity of the five-factor MSS (Hair et al., 2019; Kline, 2023).

The reliability analyses likewise demonstrated satisfactory psychometric performance. The internal consistency of the subscale coefficients ranged from 0.80 to 0.93, indicating satisfactory to excellent internal consistency. Among the five dimensions, Self-Observation demonstrated the highest reliability (α = 0.93), whereas Environmental Observation yielded the lowest, although still acceptable, reliability coefficient (α = 0.80). According to commonly accepted psychometric criteria, reliability coefficients exceeding 0.70 indicate acceptable internal consistency (Hair et al., 2019). Comparable reliability estimates have also been reported for the Five Facet Mindfulness Questionnaire (FFMQ) across different cultural contexts (Baer et al., 2006; Cebolla et al., 2012; Manotas et al., 2014; Williams et al., 2014). The omega and composite reliability coefficients further supported the internal consistency of the MSS. Taken together, these findings indicate that the Mindfulness in School Scale (MSS) demonstrates satisfactory psychometric properties across its five dimensions.

### 4.3. General Discussion

This study employed an exploratory sequential mixed methods design to develop and validate the Mindfulness in School Scale (MSS) while simultaneously providing a contextually grounded understanding of mindfulness in school settings. The general discussion therefore integrates the qualitative and quantitative findings, highlights the contribution of the environmental dimensions of mindfulness identified in this study, considers the contextual factors that may explain differences from previous research, and outlines the study’s limitations.

One of the principal findings of the study is the strong convergence between the qualitative and quantitative phases. Because the MSS was developed directly from the phenomenological findings, the factor structure obtained through the quantitative analyses largely reflected the conceptual framework generated during the qualitative phase. This convergence provides support for the exploratory sequential mixed methods design and demonstrates that the scale captures dimensions of mindfulness that are grounded in teachers’ lived experiences rather than relying exclusively on existing theoretical models. Nevertheless, one noteworthy discrepancy emerged between the two phases. Although Reactive Control appeared as a distinct theme during the qualitative analysis, it did not emerge as an independent factor in the psychometric analyses.

The absence of Reactive Control as a separate dimension appears theoretically meaningful rather than problematic. Conceptually, reactive control and acceptance represent closely related components of mindfulness. Whereas Acceptance primarily reflects individuals’ cognitive and emotional responses to unpleasant experiences, Reactive Control concerns the regulation of behavioral responses to such experiences (Baer et al., 2008). Previous mindfulness instruments have similarly differed in the extent to which these constructs are distinguished. For example, the Cognitive and Affective Mindfulness Scale–Revised (CAMS-R) conceptualizes reactive control as part of acceptance rather than as a separate construct (Feldman et al., 2007). The findings of the present study therefore suggest that, within school settings, reactive control may function as a behavioral expression of acceptance rather than as an independent dimension of mindfulness (Baer et al., 2008; Kalill et al., 2014).

A second major contribution of this study concerns the identification of Environmental Observation and Environmental Awareness as core dimensions of mindfulness in school settings. The findings indicate that mindfulness among teachers is shaped not only by intrapersonal processes but also by continuous interactions with students, colleagues, parents, and the broader organizational environment. This observation supports previous conceptualizations of interpersonal mindfulness, which emphasize awareness of both oneself and others during social interactions (Frank et al., 2016; Pratscher et al., 2018). Although interpersonal mindfulness has increasingly received attention within educational leadership research (Doornich & Lynch, 2024), relatively few psychometric instruments explicitly incorporate environmental awareness as a distinct component of mindfulness. The present findings therefore extend existing conceptualizations by demonstrating that mindfulness within schools is inherently relational and context-dependent.

This interpretation is also consistent with evidence from cognitive neuroscience and organizational psychology. Mindfulness has been shown to enhance attentional regulation while simultaneously improving individuals’ capacity to process relevant environmental information (Ji et al., 2026; Malinowski, 2013). Such attentional flexibility enables individuals to alternate efficiently between rapidly changing situational demands and sustained goal-directed attention (Farb et al., 2007; Rupprecht et al., 2019), a competency that is particularly valuable in contemporary educational environments characterized by constant interruptions, competing responsibilities, and high cognitive demands (Ehrlich, 2015). Similar to organizational leaders, teachers are expected to make rapid decisions, manage multiple interpersonal interactions, and maintain effective instructional practices while coping with substantial occupational stress (S. Johnson et al., 2005; Roeser et al., 2012; West et al., 2001). Despite these demands, they are expected to remain attentive, emotionally regulated, and responsive to students’ needs (Farb et al., 2014; Frank et al., 2016; Roeser et al., 2012; Schutz & Zembylas, 2009; Zapf, 2002). The findings of the present study suggest that environmental observation and environmental awareness may facilitate these processes by enabling teachers to recognize others’ emotional states, regulate automatic reactions, and respond more adaptively during stressful interpersonal situations. Consequently, environmental dimensions of mindfulness may represent particularly important competencies within educational settings (Barnes et al., 2007; Bihari & Mullan, 2014; Frank et al., 2016; Huston et al., 2011; Á. I. Langer et al., 2017; Wachs & Cordova, 2007).

Beyond providing a context-specific measurement instrument, the present findings suggest that mindfulness in school settings may be conceptualized as a fundamentally relational phenomenon rather than a purely intrapersonal capacity. Although traditional conceptualizations of mindfulness primarily emphasize individuals’ awareness of their internal thoughts, emotions, and present-moment experiences (e.g., Baer et al., 2006; Brown & Ryan, 2003), the qualitative and quantitative findings of this study indicate that teachers’ mindfulness is enacted through continuous interactions with students, colleagues, parents, and the broader organizational environment. In this regard, Environmental Observation and Environmental Awareness should not be interpreted merely as indicators of empathy or professional competence. Rather, they represent organizational forms of mindful attention through which teachers consciously monitor interpersonal dynamics, institutional expectations, and contextual conditions while making professional judgments. Accordingly, mindfulness in schools appears to emerge not only from awareness of the self but also from sustained engagement with the social and organizational realities of educational institutions. This perspective extends the existing mindfulness literature by suggesting that organizational context may play an important role in how mindfulness is experienced and enacted in educational practice.

The findings also contribute to the growing literature demonstrating that mindfulness may manifest differently across cultural and professional contexts (Bravo et al., 2016, 2018; Sahdra et al., 2017). Braun et al. (2024), for example, suggested that teachers’ experiences of mindfulness may vary across national contexts. Similarly, previous studies have emphasized that mindfulness in schools is a multidimensional framework comprising related but distinct dimensions whose expression is influenced by contextual, organizational, and cultural factors (Baelen et al., 2023; Renshaw & Phan, 2023). These findings underscore the importance of adapting mindfulness measures not only to specific populations but also to the educational environments in which they are intended to be used (Bergomi et al., 2013). Within this context, the present study provides evidence from Türkiye, a non-Western developing country characterized by relatively bureaucratic and hierarchical organizational structures within its educational system (Kılınç et al., 2024). The dimensions identified in this study may therefore reflect both broader characteristics of mindfulness and organizational features of the Turkish school context.

The cultural and organizational characteristics of Turkish schools may also have contributed to the particular factor structure identified in the MSS. The relatively bureaucratic and hierarchical organization of the Turkish educational system may heighten teachers’ attention to institutional expectations, organizational conditions, interpersonal roles, and the needs of different educational stakeholders, potentially contributing to the emergence of Environmental Observation and Environmental Awareness as distinct dimensions. Similarly, culturally and organizationally shaped norms concerning emotional expression, interpersonal relationships, and responses to workplace challenges may have influenced the empirical relationship between Acceptance and Reactive Control. These interpretations should, however, be regarded as contextually grounded theoretical explanations rather than empirically tested cultural effects. Whether the five-factor structure identified in the present study is specific to the Turkish educational context or generalizes across educational systems with different cultural and organizational characteristics remains to be established through cross-cultural validation. Accordingly, the MSS contributes to the growing body of cross-cultural mindfulness research by demonstrating that the expression and measurement of mindfulness should be understood within their broader sociocultural and institutional contexts.

## 5. Limitations and Future Directions

Several limitations should be considered when interpreting the findings. First, the quantitative phase relied exclusively on self-report data collected from the same respondents at a single measurement occasion. Consequently, responses may have been influenced by social desirability and other response biases. This concern may be particularly relevant to the Self-Observation dimension, which exhibited relatively high scores and a potential ceiling effect. Although Harman’s single-factor test indicated that a single factor did not account for the majority of the variance, common method variance cannot be completely ruled out. Second, the quantitative samples were recruited through convenience sampling from public schools in eastern Türkiye, particularly Kars and Iğdır provinces. Accordingly, the sample should not be considered nationally representative of teachers in Türkiye, and the findings may not generalize to teachers working in private schools, other geographical regions, or different cultural and educational contexts. Third, although the MSS demonstrated evidence of structural, convergent, and discriminant validity within the present data, convergent validity was not examined against an established external mindfulness measure such as the FFMQ or MAAS. Therefore, the extent to which the MSS dimensions correspond to established measures of mindfulness remains to be determined. Fourth, although the qualitative phase relied on in-depth interviews and multiple procedures to enhance trustworthiness, the quantitative validation was based primarily on cross-sectional self-report evidence. Finally, because the MSS dimensions demonstrated relatively low intercorrelations, the instrument should be interpreted at the subscale level rather than through a single overall score.

Future research should validate the MSS using larger and more geographically diverse samples, including teachers from different regions, educational settings, and institutional contexts. Cross-cultural replication is also needed to determine whether the five-factor structure generalizes beyond the Turkish educational context, supported by measurement invariance analyses across countries and cultural groups. Further validation should examine relationships between the MSS and established mindfulness measures, such as the FFMQ and MAAS, as well as theoretically related constructs including teacher well-being, emotion regulation, self-compassion, occupational stress, and professional functioning. Given the potential influence of socially desirable responding, future studies should also incorporate an established social desirability measure, such as the Marlowe–Crowne Social Desirability Scale. Longitudinal, multi-source, and observational designs would further reduce reliance on single-source self-report data and provide stronger evidence regarding the predictive and criterion-related validity of the MSS. In addition, future research could use latent profile analysis to investigate whether distinct mindfulness profiles emerge among teachers and whether such profiles help explain the weak relationships observed between Acceptance and some observation-related dimensions.

## 6. Implications

Mindfulness in School Scale (MSS) also has several practical implications for educational research and practice. As a context-specific instrument, the MSS may assist schools and educational authorities in identifying teachers’ strengths and developmental needs across multiple dimensions of mindfulness. The scale can be used to inform the design and evaluation of professional development programs aimed at enhancing teachers’ and school administrators’ reflective practices, emotional awareness, and responsiveness to the school environment. Furthermore, the MSS may serve as a valuable outcome measure for evaluating the effectiveness of mindfulness-based interventions and teacher well-being initiatives by assessing changes in teachers’ mindfulness over time. School leaders may also use the instrument to better understand organizational conditions that support or hinder mindful professional practice, thereby informing strategies to promote healthier school climates, collaborative relationships, and teacher well-being. Consequently, the MSS provides not only a psychometrically sound assessment tool but also a practical resource for supporting evidence-based decision-making and continuous professional learning within school settings.

From a theoretical perspective, the findings may also have implications for emotional intelligence research. Several dimensions identified in the MSS, including self-observation, environmental observation, environmental awareness, and acceptance, can be conceptually related to competencies emphasized in contemporary models of emotional intelligence, such as emotional self-awareness, empathy, emotion regulation, and adaptive interpersonal functioning (Mayer et al., 2016). However, the MSS was developed to assess mindfulness rather than emotional intelligence, and emotional intelligence was not directly measured in the present study. Therefore, these potential connections should be regarded as theoretical rather than as empirically established relationships. The multidimensional structure of the MSS may nevertheless provide a context-specific framework for future research examining whether and how mindfulness is associated with emotionally intelligent functioning among teachers. Such research should directly examine the relationships between MSS dimensions and established measures of emotional intelligence and investigate how these distinct but potentially related constructs contribute to teachers’ professional functioning and well-being.

## Figures and Tables

**Table 1:** Themes, Categories, and Representative Quotations Derived From the Qualitative Analysis.

Themes	Categories	Representative Quotations
Self-Observation	Behavioral observation experiences	“Our behaviors here are somewhat more formal and operate within institutional rules and procedures.”
	Emotional observation experiences	“My emotions generally define my day.”
Environmental Observation	Empathy experiences	“You put yourself in the child’s place.”
	Observation experiences in the school environment	“I pay particular attention to issues… that concern external stakeholders.”
Self-Awareness	Self-awareness experiences	“The responses lead me toward greater self-awareness.”
	Perceived effects of self-awareness	“I am aware of it, and I try to resolve it within myself.”
	Experiences that hinder self-awareness	“Because of the workload... I cannot fully focus on my lessons.”
Environmental Awareness	Awareness of school stakeholders	“There are some students whose potential you can clearly recognize. You are aware of it.”
	Professional awareness experiences	“After all, we carry the responsibilities of the principal’s role.”
Acceptance	Acceptance experiences	“It is important for me to acknowledge my mistakes as soon as possible.”
	Experiences of non-acceptance	“It stays on our minds—it definitely stays with us.”
Reactive Control	Experiences of controlling reactions	“To reduce the conflict at that moment, we choose to step away.”
	Experiences of expressing reactions	“If an argument occurs at school, I get angry. I do express my anger.”

**Table 2:** Factor Structure of the Mindfulness in School Scale (MSS): Exploratory and Confirmatory Factor Analysis Results.

Factors	MSS Items	Factor Loadings	
		EFA	CFA
Self-Observation	1-I pay attention to the words I use at school.	0.66	0.62
	2-I behave carefully in front of students at school.	0.89	0.83
	3-I pay attention to my behavior in front of teachers at school.	0.81	0.89
	4-I pay attention to my behavior in front of parents at school.	0.84	0.79
Environmental Observation	5-I understand the feelings of the parents at my school.	0.54	0.57
	6-I understand the feelings of the students at my school.	0.93	0.78
	7-I understand what the students at my school want and don’t want.	0.51	0.50
Self-Awareness	8-I am easily distracted at school.	0.59	0.68
	9-There are times when I feel like I am doing some things unconsciously at school without realizing it.	0.82	0.82
	10-I rush through my tasks at school without paying much attention to what I am doing.	0.57	0.72
	11-I am easily distracted while dealing with administrative tasks.	0.56	0.76
Environmental Awareness	12-I am aware of the capacity of the students at my school.	0.73	0.65
	13-I am aware of the expectations of the teachers at my school.	0.64	0.76
	14-I am aware of what can and cannot be done at school.	0.60	0.74
	15-I am aware of the current conditions of the school.	0.54	0.56
Acceptance	16-I cannot forget a bad thing that happened to me at school.	0.51	0.57
	17-Undesirable situations at school make me feel fed up.	0.88	0.90
	18-If I experience something negative at school, I will become disillusioned with school.	0.66	0.79

**Table 3:** Confirmatory Factor Analysis Fit Indices for the Mindfulness in School Scale (MSS).

Fit Index	Recommended Values	Observed Values
X^2^	Lower values indicate better fit	150.331
df	—	125
X^2^/df	<3 acceptable	1.20
SRMR	<0.08 acceptable	0.047
RMSEA	<0.08 acceptable; <0.06 good	0.028
RMSEA 90% CI	—	[0.000, 0.043]
GFI	≥0.90 acceptable; ≥0.95 good	0.944
AGFI	≥0.90 acceptable	0.923
NFI	≥0.90 acceptable; ≥0.95 good	0.914
TLI	≥0.90 acceptable; ≥0.95 good	0.981
CFI	≥0.90 acceptable; ≥0.95 good	0.984

Note. CFA was estimated using conventional Maximum Likelihood (ML) estimation. χ^2^ = chi-square; df = degrees of freedom; SRMR = Standardized Root Mean Square Residual; RMSEA = Root Mean Square Error of Approximation; CI = confidence interval; GFI = Goodness-of-Fit Index; AGFI = Adjusted Goodness-of-Fit Index; NFI = Normed Fit Index; TLI = Tucker–Lewis Index; CFI = Comparative Fit Index.

**Table 4:** Reliability Estimates for the Mindfulness in School Scale (MSS).

Factors	Cronbach’s Alfa (α)	McDonald’s Omega (ω)	Composite Reliability (CR)	Corrected Item–Total Correlations
1. Self Observation	0.93	0.94	0.832	0.654–0.793
2. Environmental Observation	0.80	0.83	0.704	0.476–0.606
3. Self Awareness	0.84	0.86	0.776	0.496–0.625
4. Environmental Awareness	0.86	0.87	0.790	0.519–0.567
5. Acceptance	0.83	0.86	0.805	0.446–0.575

**Table 5:** Descriptive Statistics and Intercorrelations Among the MSS Subscales.

Factors	Mean	SD	Skewness	Kurtosis	1	2	3	4	5
1. Self-Observation	4.73	0.40	−0.973	−0.638	-				
2. Environmental Observation	4.30	0.46	0.040	−0.211	0.30 **	-			
3. Self-Awareness	4.44	0.42	−0.478	0.151	0.18 **	0.11 *	-		
4. Environmental Awareness	4.01	0.68	−1.125	1.382	0.44 **	0.41 **	0.17 **	-	
5. Acceptance	2.75	0.88	0.020	−0.621	0.01	−0.08	0.31 **	−0.14 **	-

Note. * indicates *p* < .05; ** indicates *p* < .001.

**Table 6:** Convergent and Discriminant Validity of the MSS Using the Fornell–Larcker Criterion.

Factors	AVE	1	2	3	4	5
1. Self-Observation	0.622	**0.789**				
2. Environmental Observation	0.394	0.30	**0.628**			
3. Self-Awareness	0.558	0.18	0.11	**0.747**		
4. Environmental Awareness	0.465	0.44	0.41	0.17	**0.682**	
5. Acceptance	0.586	0.01	−0.08	0.31	−0.14	**0.766**

Note. AVE = Average Variance Extracted. Bold diagonal values represent the square root of AVE (√AVE), whereas off-diagonal values represent correlations among the MSS dimensions.

**Table 7:** Measurement Invariance of the MSS Across Gender.

	Model Fit						Model Comparison	
Gender (male/female)	χ^2^	df	*p*	CFI	TLI	RMSEA	ΔCFI	ΔRMSEA
Configural	294.799	250	.027	0.973	0.967	0.037	—	—
Metric	313.665	263	.017	0.970	0.965	0.038	−0.004	0.001
Scalar	333.743	276	.010	0.965	0.962	0.040	−0.004	0.002

Note. χ^2^ = chi-square; *df* = degrees of freedom; CFI = Comparative Fit Index; TLI = Tucker–Lewis Index; RMSEA = root mean square error of approximation; ΔCFI = change in CFI; ΔRMSEA = change in RMSEA. Changes in fit indices for metric and scalar invariance are relative to the immediately preceding model.

## Data Availability

The data presented in this study are available from the corresponding author upon reasonable request. The data are not publicly available due to ethical and privacy considerations involving research participants.

## Supplementary Materials

The following supporting information can be downloaded at: https://www.mdpi.com/article/10.3390/jintelligence14090210/s1, Supplementary File S1: Mindfulness in School Scale (MSS)—Ready-to-Use Instrument.
