# Supplementary material for: Conceptualizing and Measuring Mindfulness in School Settings: A Mixed Methods Study and Development of Mindfulness in School Scale
Source: J Intell. 2026 Sep 1;14(9):210. doi: 10.3390/jintelligence14090210 (PMC13608719; doi:10.3390/jintelligence14090210)
Supplement: Supplementary file 1 [file jintelligence-14-00210-s001.zip › jintelligence-4489600-supplementary.pdf]

Mindfulness in School Scale (MSS): Ready-to-Use Instrument

The Mindfulness in School Scale (MSS) is an 18-item instrument designed to assess mindfulness within school settings across five dimensions: **Self-Observation, Environmental Observation, Self-Awareness, Environmental Awareness, and Acceptance**. The original Turkish version of the MSS was developed and psychometrically evaluated in the present study. Scores should be calculated and interpreted separately for each dimension rather than as a single total score.

Original Turkish Version

Okullarda Bilinçli Farkındalık Ölçeği

Yönerge. Aşağıdaki ifadelere vereceğiniz cevapları, **1 = Hiçbir zaman, 2 = Nadiren, 3 = Bazen, 4 = Çoğu zaman ve 5 = Her zaman** seçeneklerinden size en uygun olanını (X) ile işaretleyerek belirtiniz. İfadelerin doğru veya yanlış cevabı yoktur. Bu nedenle, her ifadeyi okuduğunuzda aklınıza gelen ilk cevabı vermeniz önerilmektedir.

No.	Madde	1 Hiçbir zaman	2 Nadiren	3 Bazen	4 Çoğu zaman	5 Her zaman
**Öz-Gözlem**						
1	Okulda kullandığım sözlere dikkat ederim.	☐	☐	☐	☐	☐
2	Okulda öğrencilerin karşısında dikkatli davranırım.	☐	☐	☐	☐	☐
3	Okulda öğretmenlerin yanında davranışlarıma dikkat ederim.	☐	☐	☐	☐	☐
4	Okulda velilerin yanında davranışlarıma dikkat ederim.	☐	☐	☐	☐	☐
**Çevresel Gözlem**						
5	Okulumdaki velilerin hislerini anlarım.	☐	☐	☐	☐	☐
6	Okulumdaki öğrencilerin hislerini anlarım.	☐	☐	☐	☐	☐
7	Okulumdaki öğrencilerin neyi isteyip neyi istemediklerini anlarım.	☐	☐	☐	☐	☐
**Öz-Farkındalık**						
8	Okulda dikkatim kolay dağılır.	☐	☐	☐	☐	☐
9	Okulda farkına varmaksızın bazı şeyleri bilinçsizce yapıyormuşum gibi hissettiğim zamanlar olur.	☐	☐	☐	☐	☐

No.	Madde	1 Hiçbir zaman	2 Nadiren	3 Bazen	4 Çoğu zaman	5 Her zaman
10	Okulda ne yaptığıma çok dikkat etmeksizin işlerimi acele ile yapıp geçerim.	☐	☐	☐	☐	☐
11	İdari işler ile uğraşırken dikkatim kolay dağılır.	☐	☐	☐	☐	☐
**Çevresel Farkındalık**						
12	Okuldaki öğrencilerin kapasitesinin farkındayım.	☐	☐	☐	☐	☐
13	Okuldaki öğretmenlerin isteklerinin farkındayım.	☐	☐	☐	☐	☐
14	Okulda yapılabilecek ve yapılamayacak şeylerin farkındayım.	☐	☐	☐	☐	☐
15	Okulun mevcut şartlarının farkındayım.	☐	☐	☐	☐	☐
**Kabullenme**						
16	Okulda başıma gelen kötü bir şeyi unutamam.	☐	☐	☐	☐	☐
17	Okulda istenmeyen durumlar olması bende bıkkınlık yaratır.	☐	☐	☐	☐	☐
18	Okulda olumsuz bir şey yaşarsam okula karşı soğurum.	☐	☐	☐	☐	☐

Scoring of the Original Turkish MSS

The five subscales are scored separately:

Self-Observation: Items 1–4
Environmental Observation: Items 5–7
Self-Awareness: Items 8–11
Environmental Awareness: Items 12–15
Acceptance: Items 16–18

Items **8-9-10–11 and 16-17–18 are negatively worded** relative to their respective construct labels and should be **reverse-scored** before calculating the corresponding subscale scores:

1 → 5, 2 → 4, 3 → 3, 4 → 2, 5 → 1.

A subscale score can be calculated as the **mean of the items belonging to that dimension**. Higher scores indicate higher levels of the corresponding mindfulness dimension. Because the five MSS dimensions showed relatively low intercorrelations in the present validation study, **calculation and interpretation of a single overall MSS score are not recommended**.

English Translation

Mindfulness in School Scale (MSS)

Instructions. Please indicate how frequently each of the following statements reflects your experiences and behaviors in the school setting. Select one response for each statement using the following scale: **1 = Never, 2 = Rarely, 3 = Sometimes, 4 = Often, and 5 = Always**. There are no right or wrong answers.

No.	Item	1 Never	2 Rarely	3 Sometimes	4 Often	5 Always
**Self-Observation**						
1	I pay attention to the words I use at school.	☐	☐	☐	☐	☐
2	I behave carefully in front of students at school.	☐	☐	☐	☐	☐
3	I pay attention to my behavior in front of teachers at school.	☐	☐	☐	☐	☐
4	I pay attention to my behavior in front of parents at school.	☐	☐	☐	☐	☐
**Environmental Observation**						
5	I understand the feelings of the parents at my school.	☐	☐	☐	☐	☐
6	I understand the feelings of the students at my school.	☐	☐	☐	☐	☐
7	I understand what the students at my school want and don't want.	☐	☐	☐	☐	☐
**Self-Awareness**						
8	I am easily distracted at school.	☐	☐	☐	☐	☐
9	There are times when I feel like I am doing some things unconsciously at school without realizing it.	☐	☐	☐	☐	☐
10	I rush through my tasks at school without paying much attention to what I am doing.	☐	☐	☐	☐	☐
11	I am easily distracted while dealing with administrative tasks.	☐	☐	☐	☐	☐

No.	Item	1<br>Never	2<br>Rarely	3<br>Sometimes	4<br>Often	5<br>Always
**Environmental Awareness**						
12	I am aware of the capacity of the students at my school.	☐	☐	☐	☐	☐
13	I am aware of the expectations of the teachers at my school.	☐	☐	☐	☐	☐
14	I am aware of what can and cannot be done at school.	☐	☐	☐	☐	☐
15	I am aware of the current conditions of the school.	☐	☐	☐	☐	☐
**Acceptance**						
16	I cannot forget a bad thing that happened to me at school.	☐	☐	☐	☐	☐
17	Undesirable situations at school make me feel fed up.	☐	☐	☐	☐	☐
18	If I experience something negative at school, I will become disillusioned with school.	☐	☐	☐	☐	☐

Scoring

Self-Observation: Items 1–4
Environmental Observation: Items 5–7
Self-Awareness: Items 8–11
Environmental Awareness: Items 12–15
Acceptance: Items 16–18

Items **8–11 and 16–18 should be reverse-scored** (1 = 5, 2 = 4, 3 = 3, 4 = 2, and 5 = 1). Subscale scores can then be calculated by averaging the items within each dimension. Higher scores indicate higher levels of the corresponding dimension.

A **total MSS score is not recommended**; the five subscales should be interpreted separately.

Note. The Turkish version is the original version of the MSS developed and psychometrically validated in the present study. The English version is provided to facilitate international accessibility and has not yet been independently psychometrically validated. Researchers intending to administer the English version should evaluate its psychometric properties in the target population.
